# Experiences and Perceptions of Medication Management Communication During Transitions of Care for Residents in Aged Care Homes and Their Caregivers: A Qualitative Meta‐Synthesis

**DOI:** 10.1111/jocn.17438

**Published:** 2024-10-06

**Authors:** Alison Dowling, Stephanie Garratt, Elizabeth Manias

**Affiliations:** ^1^ School of Nursing and Midwifery Monash University Clayton Victoria Australia

**Keywords:** aged care, communication, family caregivers, medication therapy management, person‐centred care, residents, transitions of care

## Abstract

**Aim:**

To explore the experiences and perceptions of communication about managing medication across transitions of care for residents living in aged care homes and their family caregivers.

**Background:**

Effective medication communication across transitions of care involves exchanging information, resident, and family caregiver's participation in decision‐making, and shared responsibility.

**Design:**

A qualitative meta‐synthesis.

**Method:**

This review was conducted in accordance with the PRISMA 2020 guidelines and the accompanying 27‐item checklist. A systematic search of seven electronic databases (Embase, PsycINFO, Medline Ovid, Scopus, CINAHL, EmCare and Web of Science) was performed from inception to December 2023. Studies eligible for inclusion in this review were required to be published in peer‐reviewed English journals and focus on medication communication among healthcare providers, residents and family caregivers during transitions of care for aged care residents. The JBI Critical Appraisal Checklist for Qualitative Research was employed for the critical appraisal of the studies, and the COREQ checklist was used to evaluate their quality.

**Results:**

Of the 2610 studies identified, 12 met the inclusion criteria. No study was excluded based on quality. Two main themes were generated: (1) Medication information exchange involving residents and families, and (2) resident and family factors influencing medication communication engagement. The findings revealed a lack of supportive structure for effective communication and collaboration among residents, family caregivers and healthcare providers during transitions of care, marked by one‐way interactions and limited evidence of shared decision‐making or family caregiver engagement in medication management communication, despite varying individual needs and preferences.

**Conclusions:**

Communication about medication management during transitions of care focused on sharing details rather than active engagement. Residents and their family caregivers have individual needs and perspectives regarding communication about medication management, which are not well addressed by healthcare providers during transitions of care. Healthcare providers' communication remains limited, and family caregivers are underutilised.

**Summary**

- What this paper contributes to the wider clinical community?
  ○ The findings show there is lack of research on communication about medication management across transitions of care for residents and their family caregivers.
  ○ Lacking insights into the specific medication communication needs of residents and their family caregivers has led to communication that overlooks their unique perspectives and requirements, failing to adopt a person‐centred approach.
  ○ By acknowledging and addressing these gaps diverse strategies can be formulated and implemented to bridge these divides and ultimately enhance resident outcomes throughout the transitions of care process.

## 1 Introduction

Challenges are occurring on a global level, posed by an ageing population that is living at home longer (Elliott and Booth 2014). Consequently, aged care homes are providing care for individuals who are increasingly older and more medically complex (Elliott and Booth 2014).

People with multiple medical conditions are more likely to take many medications, and this places them at risk of experiencing medication errors (Desai et al. 2011; Duerden, Avery, and Payne 2013) and adverse drug events (Crotty et al. 2005; Elliott and Booth 2014), often leading to hospitalisations (Barber et al. 2009). As a result, people living in aged care settings are more likely to experience frequent transitions of care to hospital, and these transitions are likely to be more complex (Chhabra et al. 2012). ‘Transitions of care’, when residents move between healthcare settings and providers (Coleman et al. 2004), are vulnerable points for medication issues due to potential communication breakdowns during activities such as handovers, admission and discharge consultations between healthcare providers, residents and family caregivers (World Health Organization 2016).

Transitions between care settings can introduce risk of resident harm through poor communication and unintended or unnecessary changes to medications (Redmond et al. 2018). On average, residents experience five to seven medication changes during hospitalisation, with two to three medications discontinued and three to four medications started (Elliott 2006). These changes are not always communicated appropriately to the resident, family caregivers or primary healthcare providers, which can lead to incorrect medicine use and inappropriate management of residents' conditions (Daliri et al. 2019).

Studies report varied medication error rates (16%–27%) during transitions between hospitals and aged care homes, with approximately 17% of residents experiencing adverse drug events in the post‐hospital discharge phase (Ferrah, Lovell, and Ibrahim 2017; Kapoor et al. 2019). The financial burden of medication‐related harm among the resident population is high and is expected to escalate as aged care homes face increased demand and care for individuals with complex needs (Jokanovic et al. 2019; Manias et al. 2021).

Ensuring timely, accurate and person‐family‐centred medication handover between healthcare providers, residents and family caregivers is pivotal to reducing serious medication errors and harm during transitions of care (Wheeler et al. 2018). Since residents and their family caregivers serve as the common link across different providers and settings (Coleman et al. 2006), it follows that any attempt to improve communication across transitions of care would, by necessity, involve resident and family carer engagement in order to achieve a person‐centred focus. Given the risks and challenges associated with medication management during transitions of care, it is important to understand the needs and preferences of both groups so that strategies can be designed to support and respond to these needs. Effective medication communication relies on information exchange, resident, and family involvement in decision‐making, and shared responsibility (Manias 2010).

Previous research highlights significant challenges in communicating medication information to aged care home residents and their caregivers (Bauer, Fitzgerald, and Koch 2011; Hesselink et al. 2012; McGilton et al. 2017). Cognitive impairments, such as dementia, complicate understanding and processing spoken instructions (McGilton et al. 2017), while sensory impairments like hearing loss further hinder effective communication (Erber and Scherer 1999). Language barriers and varying health literacy levels can lead to misunderstandings (Joseph‐Williams, Elwyn, and Edwards 2014). Low health literacy and vision impairments also make reading medication lists and care plans difficult (Shahid et al. 2022). Caregivers often find the complexity and volume of information overwhelming, especially during care transitions (Bauer, Fitzgerald, and Koch 2011; Hesselink et al. 2012). Effective communication is further challenged by logistical issues in transferring information between care settings (Bauer, Fitzgerald, and Koch 2011; Coleman et al. 2004; Hesselink et al. 2012), inadequate staffing, heavy workloads and lack of training (McGilton et al. 2017; Sawan et al. 2017; Stacey, Samant, and Bennett 2008). These factors underscore the need for better communication strategies, especially during transitions of care (Coleman 2003; Crilly, Chaboyer, and Wallis 2006).

Previous systematic reviews have primarily examined engagement in medication management during transitions of care from the perspectives of older patients transitioning between their homes and hospitals, along with insights from their family caregivers (Manias et al. 2020; Ozavci et al. 2021; Tobiano et al. 2019). Some prior studies have also explored healthcare provider perspectives on resident movements between aged care homes and hospitals, with minimal emphasis on medication communication (Pulst et al. 2020; Stephens et al. 2015; Tsai, Tsai, and Huang 2016). The findings from these studies have highlighted that communication regarding medication management during transitions of care involving older patients and family. Examining these aspects within the resident population is crucial to mitigate the risk of medication errors, adverse events and ultimately harm during transitions of care.

To address the knowledge gap, this qualitative meta‐synthesis aims to explore the experiences and perceptions of communication about medication management across transitions of care for aged care facility residents and their family caregivers. It is anticipated this information will highlight opportunities for improving healthcare provider, resident and family engagement in medication communication during transitional care.

### 1.1 Aims

To explore the experiences and perceptions of communication about medication management across transitions of care for aged care facility residents and their family caregivers. The research question is: During transitions of care, what are the experiences and perceptions of aged care home residents and their family caregivers regarding communication on medication management provided by healthcare providers?

In this review, medication communication involves sharing and understanding information about medications, including decision‐making facilitation, preferences and care goals among residents, family caregivers and healthcare providers. This communication can occur through various channels such as verbal, non‐verbal, written or electronic means. Examples include discussions about medication regimens and the exchange of medication‐related documentation.

## 2 Methods

### 2.1 Design

This is a systematic evaluation and meta‐synthesis of qualitative research.

### 2.2 Search Strategy

This qualitative meta‐synthesis was undertaken in accordance with the guidelines outlined in the Preferred Reporting Items for Systematic Reviews and Meta‐Analyses (PRISMA) statement (Page et al. 2021), and the review is reported using the 27‐item PRISMA checklist (Page et al. 2021) (see Appendix S1). A systematic search was conducted across seven databases from inception to December 2023 using Embase, PsycINFO, Medline Ovid, Scopus, CINAHL, EmCare and Web of Science. There were no limitations on the year of publication. To ensure a comprehensive search strategy, a subject specialist librarian with expertise in systematic review expertise from the university assisted in developing the search strategy. Four groups of key terms were searched individually and then combined using Boolean operators ‘OR’ and ‘AND’. These four groups were (1) aged, elderly, caregiver, family; (2) communication, polypharmacy, medication errors, drug utilisation review, deprescription, medication utilisation review, medication management; (3) transitional care, transitions of care, transfer; (4) nursing home, long‐term care, homes for the aged, emergency service, emergency medical services, patient discharge. See Appendix S2 for the search terms and search results. The search results were imported into EndNote X8 (Clarivate Analytics) and then subsequently imported into Covidence Systematic Review Management Software (Veritas Health Innovation 2021), where duplicate records were removed, and screening and data extraction were conducted. Two independent reviewers (A1 and A2) assessed the relevancy of study titles and abstracts based on the inclusion criteria, with a third reviewer (A3) resolving any discrepancies. Papers deemed relevant at the title and abstract level underwent eligibility assessment at the full‐text level by two independent reviewers (A1 and A2). Any discrepancies during the full‐text screening process were resolved by a third reviewer (A3).

### 2.3 Inclusion and Exclusion Criteria

#### 2.3.1 Inclusion Criteria

Studies meeting the following criteria were included in the review: published in peer‐reviewed journals and in English, any publication date and focused on communication between residents, family caregivers and healthcare providers regarding medication management during transitions of care. The phenomenon of interest was medication communication, which included exchanging information about medications among residents, family caregivers and healthcare providers, or participating in medication‐related decision‐making. This communication involved verbal, non‐verbal, written or electronic interactions. Participants included individuals living permanently in aged care homes and/or family caregivers, aged 18 years and over. In addition, data had to be collected directly from residents and/or family caregivers.

#### 2.3.2 Exclusion Criteria

Studies were excluded from the review if they were non‐research studies, such as comments, letters, editorials and conference abstracts. Additionally, unpublished literature, research protocols, literature reviews and case reports were also excluded. Studies involving non‐permanent aged care home settings, such as home care, community care, skilled nursing facilities and rehabilitation/transitional care facilities, were also excluded.

### 2.4 Data Synthesis, Extraction and Quality Appraisal

Data extracted from the results sections of the included studies were entered into a Microsoft Excel spreadsheet. Author/year, country, aim, methodology, participant details, sample size, settings and key findings were extracted. Data were extracted at the level of themes. Data were extracted from each paper by two reviewers independently (A1 and A3). The data analysis and synthesis were conducted by one researcher using a six‐step thematic analysis approach for qualitative research (Braun and Clarke 2006). This iterative process commenced with a thorough reading and re‐reading of the extracted data to allow familiarity and understanding. Next, initial codes were generated to identify important features and concepts. These codes were then sorted into potential categories to search for overarching themes. Following this, themes were meticulously reviewed and refined to ensure accuracy and coherence. Once the themes were clearly delineated, they were precisely defined and names, including any subthemes identified. Finally, comprehensive findings were generated by summarising and explaining the significance of each theme. To ensure rigour and reliability, the codes and themes underwent comprehensive review by a second researcher, with any discrepancies or differences in interpretation being discussed and resolved through consensus. This collaborative approach ensured the robustness and validity of the thematic analysis process. Two reviewers used the Joanna Briggs Institute (JBI) Critical Appraisal Checklist for Qualitative Research (Joanna Briggs Institute 2017) to independently assess the quality of included studies. The JBI Critical Appraisal Checklist for Qualitative Research checklist assesses the methodological rigour of qualitative research. No papers were excluded from the data synthesis based on the results obtained from the appraisal. The Consolidated Criteria for Reporting Qualitative Research (COREQ) (Tong, Sainsbury, and Craig 2007) were used to evaluate the quality of the studies. The COREQ checklist is organised into three domains: (i) research team and reflexivity, (ii) study design and (iii) data analysis and reporting (Tong, Sainsbury, and Craig 2007).

## 3 Results

### 3.1 Overview of Included Studies

**FIGURE 1:**
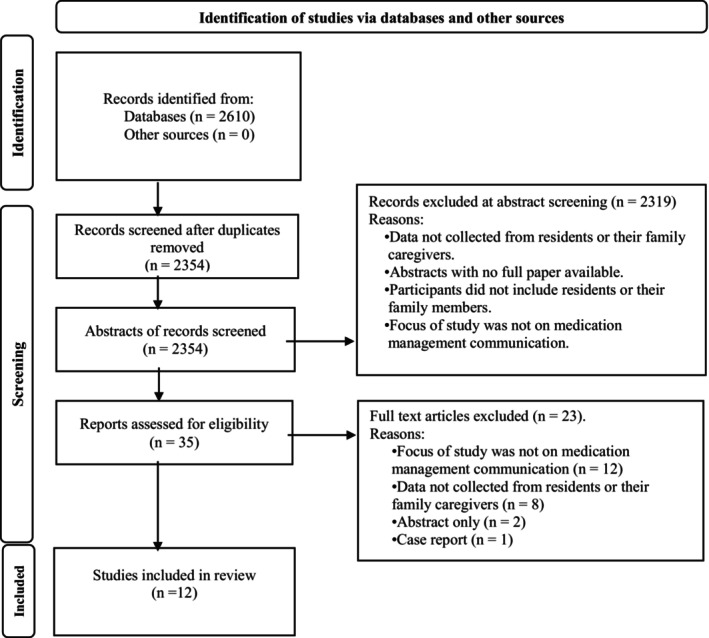
Search process flowchart (PRISMA flow diagram) (Page et al. 2021).

After conducting database searches, 2610 studies were initially retrieved, of which 12 studies met the eligibility criteria for inclusion (see Figure 1). All included studies were qualitative exploratory studies. The JBI Critical Appraisal Checklist for Qualitative Research checklist, which was used to appraise the quality of studies, had scores ranging from 1 to 10. The most common score obtained among the 12 papers was eight out of 10, with the highest score achieved being nine out of 10. See Appendix S3 for the results of the critical appraisal for all studies. The EQUATOR Consolidated Criteria for Reporting Qualitative Research (COREQ) checklist (Tong, Sainsbury, and Craig 2007) was utilised to ensure comprehensive reporting of the included qualitative research studies, following a 32‐item checklist aimed at enhancing the transparency and rigour of qualitative research (see Appendix S4 for an example the COREQ results for several of the included studies). The COREQ checklist identified a lack of detailed descriptions regarding the researchers' backgrounds and experiences across the studies examined, as well as insufficient information about study design.

### 3.2 Study Characteristics

The included studies were conducted between 2011 and 2022. They were conducted in various western countries, including Australia (5 studies), the USA (3 studies), Norway and Canada (both 2 studies apiece). In terms of data collection methods, all 12 studies utilised semi‐structured interviews. Seven studies conducted face‐to‐face interviews, three studies utilised telephone interviews and two studies a combination of both methods. Regarding study settings, 10 studies were conducted in urban‐based aged care homes, two of which also included nursing homes in rural areas. Additionally, four studies were based in urban hospitals, one in a rural hospital, and one involved community settings with family caregivers living in their own homes. The study participants included residents (4 studies), family caregivers (2 studies), or a combination of both (6 studies). Two studies focused on residents living with dementia. In total, the 12 studies represented 295 participants, with 78% being residents (229 participants) and 22% being family caregivers (66 participants). The age range for residents was from 46 to 100 years, with an average of 80 years. Family caregivers had an age range of 46 to 84 years, with an average of 63 years. The ages of family caregivers were not reported in five studies that included family caregivers as participants. Regarding sex distribution, females comprised 86% of the total resident population and 73% of the total family carer population. Besides age and sex, only one study (Sawan et al. 2021) provided additional demographic information, which is related to the education level of family caregivers (See Tables 1 and 2). Table 1 provides an overview of the combined demographic characteristics of the study participants. Table 2 provides an overview of the included studies.

**TABLE 1:** Combined demographic characteristics of participants in 12 studies.

Characteristics	Total number	Frequency (%)
Participants	349	
Residents	223	64
Family caregivers	126	36
Gender		
Female residents	189	86
Male residents	40	14
Female caregivers	48	73
Male caregivers	18	17
Age range (years)		
Residents	46–100	
Family caregivers	46–84	
Mean age (years)		
Residents	80	
Family caregivers	62	
Education		
Family caregivers		
< 12 years of education	2	6
Completed high school	3	10
Completed certificate/diploma	6	19
Bachelor's degree or above	20	65

**TABLE 2:** Characteristics of the qualitative studies.

Author/Year/Country	Methodology/Data collection	Transition of care direction	Participant characteristics	Sample size	Setting	Key findings
Abrahamson et al. (2016), USA	Qualitative exploratory study involving semi‐structured interviews (telephone)	Aged care home to hospital	Family caregivers: *n* = 20 Mean age: Not reported Females: 85%	20	Aged care homes: 9 urban	**Themes** ***Issues at hand‐off and the hospital*:** During transitions of care between aged care homes and hospital, family members described: Inadequate communication between aged care home and hospital staff about residents' medication and other needs Lack of communication with family members Lack of family consultation about medication changes made during hospitalisation
Arendts et al. (2015), Australia	Qualitative exploratory study involving semi‐structured interviews (face‐to‐face)	Aged care home to hospital	*Residents*: *n* = 11 Mean age: 88.0 years Female: 81.8% *Family members*: *n* = 14 Mean age: Not reported Female: Not reported	25	*Aged care homes*: 6 urban *Hospitals*: 1 urban	**Themes** ***Ambiguity*:** At the time of admission to the emergency department, residents and family members described: Lost medications/property during transition from the aged care home Being separated from their loved ones during consultations with healthcare staff
Canada et al. (2020), USA	Qualitative exploratory study involving semi‐structured interviews (telephone)	Aged care home‐Hospital	*Residents*: *n* = 14 Mean age 71 years (range 46–98 years) Female: Not reported	14	*Aged care homes*: 16 urban	**Themes** ***Transition back to the aged care home*:** Inconsistent medication information exchange between hospital and aged care home Inconsistent provision of medication information provided to residents at discharge
Deeks et al. (2016), Australia	Qualitative exploratory study involving semi‐structured interviews (face‐to‐face and telephone)	Aged care home‐Hospital‐Aged care home	*Carers* (*unpaid*): *n* = 4 Mean age: NR Female: NR *Carers* (*paid*): *n* = 3 Mean age: NR Female: NR	7	Settings comprised acute and primary care facilities across four sites (3 urban; 1 rural). No additional details provided	**Themes** ***Medication reconciliation at admission*:** Hospital staff had issues obtaining accurate medication information if family members absent or resident did not have medications with them ***Lack of modified planning for transitions*:** Carers received inadequate discharge medication information
Dyrstad, Laugaland, and Storm (2015), Norway	Qualitative, exploratory study involving field observations involving face‐to‐face/telephone conversations	Aged care home‐Hospital‐Aged care home	*Residents*: *n* = 9 Mean age: 83.2 years (range: 82–93 years) Female: 67% *Family members*: *n* = 6 Mean age: Not reported Female: 100%	15	*Hospitals*: 2 urban	**Themes** ***Observing professionals’ information dissemination and decision‐making*:** Lack of family participation in physicians' ward rounds to discuss medication management ***Patients’ Experiences with Integration of Information*:** Residents had problems with comprehension of medication information due to health issues Amount of information provided often overwhelming and confusing for residents ***Next of Kin Advocacy*:** Families played important advocacy roles during hospitalisation to ensure residents’ safety and continuity of care Families not routinely invited to join ward rounds with healthcare teams to discuss medication management Lack of advanced notice given to families of discharge
Gjerberg et al. (2015), Norway	Qualitative exploratory study involving semi‐structured interviews and focus groups (all face‐to‐face)	Aged care home‐Hospital‐Aged care home	*Residents*: *n* = 30 Mean age: 86.0 years (range: 68–98 years) Female: 90.0% *Family members*: *n* = 33 Mean age: Not reported Female: Not reported	68	*Aged care homes*: 6 urban	**Themes** ***Participating in decision‐making processes*:** Residents and family caregivers wanted involvement in medication decision‐making, but rarely queried decisions made by healthcare providers
McCloskey (2011), Canada	Qualitative exploratory study involving semi‐structured interviews (face‐to‐face) and observations	Aged care home‐Hospital	*Residents* (*Interviews*): *n* = 5 Mean age: 70.0 years (range 58.4–81.6) Female: 20% *Residents* (*Observations*): *n* = 61 Mean age: 80.2 (range: 67.6–92.8 years) Female: 69%	61	*Aged care homes*: 1 urban *Hospitals*: 1 urban	**Themes** ***Creating and exchanging resident information*** (a) At admission: Healthcare staff focus was on information collection, not information exchange Verbal communication among healthcare staff in different settings virtually non‐existent Ineffective use of standardised forms to share medication information during transitions; residents often tasked with filling information gaps, which was often challenging due to health issues (b) At discharge: Lack of effective processes for sharing medication information between hospital and primary care providers
Palagyi et al. (2016), Australia	Qualitative exploratory study involving focus groups and semi‐structured interviews (face‐to‐face)	Aged care home‐Hospital‐Aged care home	*Residents*: *n* = 25 Mean age: 87.6 years (range: 75–100 years) Female: 77% *Family members*: *n* = 16 Mean age: 65.3 years (range: 54–84 years) Female: 66%	41	*Aged care homes*: 3 urban	**Themes** ***Negotiating a complex system*:** Large number of new medications prescribed during hospitalisation Medication knowledge among residents and relatives: Limited medication literacy among residents and family caregivers ***Control beliefs and self‐efficacy*:** Residents and family caregivers lacked confidence in managing medication changes
Robinson et al. (2012), Canada	Qualitative exploratory study involving semi‐structured interviews (face‐to‐face)	Aged care home‐Hospital‐Aged care home	*Residents*: *n* = 7 Mean age: 79.0 years (range 72–87 years) Female: 57% *Family members*: *n* = 20 Female: 85% Mean age: Not reported	27	*Aged care homes*: 2 urban	**Themes** ***Communication of information*:** Effective medication information exchange lacking during transitions of care between aged care home and hospital; created challenges for information gathering by hospital staff. Family members often tasked with filling in information gaps Hospital healthcare providers used inconsistent methods to communicate and rarely involved residents or families in medication decision‐making ***Timeliness*:** Residents and families linked lack of discharge planning, post discharge medication supplies and communication to disruptions in continuity of care
Sawan et al. (2021), Australia	Qualitative exploratory study involving semi‐structured interviews (phone)	Hospital‐Aged care home	*Family caregivers*: *n* = 10 Mean age: 60 years (range 46–74 years) Female: 32%	10	Community (i.e., family caregivers living in their own homes)	**Themes** ***Inadequate information about medication management at discharge*:** Lack of medication information provided to family caregivers at admission & discharge Limited caregiver engagement in medication management decisions: Mixed views among caregivers regarding inclusion in medication decision‐making. Little post discharge medication management guidance provided ***Difficulties ensuring medication supply post discharge*:** Inadequate supply of discharge medications led to difficulties in ensuring on‐going supplies ***Carers overwhelmed by discharge processes*:** Carers overwhelmed by multiple and disorganised discharge processes ***Caregivers proactively seeking information to ensure avoidance of medication harm*:** Medication information required by family caregivers to increase medication management knowledge & confidence
Sawan et al. (2022), Australia	Qualitative exploratory study involving semi‐structured interviews (face‐to‐face)	Aged care home‐Hospital‐Aged care home	*Residents*: *n* = 31 Mean age: 85.0 years (range: 66–92 years) Female: 74.2%	31	*Aged care homes*: 6 urban	**Themes** ***Deferring control or participation in medication‐related decisions to others*:** Residents' low knowledge, health and comprehension influenced their participation in medication discussions; often deferred decision‐making to others Residents' beliefs about medications: Perceived harm of prescribed medications impacted residents' engagement in medication discussions ***External factors impacting residents' participation in medication discussions*:** Limited opportunities provided by healthcare providers impacted residents' participation in medication discussions
Toles et al. (2012), USA	Qualitative exploratory study involving semi‐structured interviews (face‐to‐face)	Aged care home‐Hospital‐Aged care home	*Residents*: *n* = 30 Mean age: 73.8 years Female: 77.0%	30	*Aged care homes*: 7 urban	**Themes** ***During hospitalisation*: *Limited involvement in planning with professional staff*:** Lack of engagement with residents and family caregivers in medication communication Uncertainty about hospital care and follow‐up planning: Residents and family caregivers often not informed about medication decisions and desire greater opportunities to engage with physicians

## 4 Themes

### 4.1 Medication Information Exchange Involving Resident and Families

All 12 studies explored resident and family caregiver experiences of medication communication with healthcare providers during transitions of care. This theme encompasses the perceptions, interactions and challenges encountered by residents and their family caregivers regarding medication information exchange. It includes communication during hospitalisation, discharge and post‐discharge, covering aspects such as providing and receiving medication information and involvement with healthcare providers about medication decision‐making. Sub‐themes include: (a) Consequences for residents and families as a result of information exchange practices during hospitalisation; (b) medication information exchange at discharge experienced by residents and families; and (c) effects of information exchange among healthcare staff on residents and their caregivers.

#### 4.1.1 Consequences for Residents and Families as a Result of Information Exchange Practices During Hospitalisation

All 12 studies investigated the perspectives of residents and their family members regarding communication with healthcare professionals about medications during residents' hospitalisation. For the purposes of this review, hospitalisation encompasses both emergency department and hospital ward admissions.

Upon admission to the emergency department, communication primarily involved healthcare providers, such as nurses, physicians and pharmacists, seeking medication information from residents and family caregivers. This was challenging for most residents due to their heightened anxiety, confusion, poor physical health and cognitive issues (Dyrstad, Laugaland, and Storm 2015). Some residents lacked understanding about their medications and were not confident about providing details about their medications to healthcare providers (Palagyi et al. 2016). In cases where family caregivers were absent or residents arrived without medications, challenges in verifying medications were heightened (Robinson et al. 2012). In addition, observations revealed that families were often separated from residents during triage assessments, which restricted communication between healthcare teams and family caregivers (Arendts et al. 2015). Some family caregivers also believed they had minimal knowledge about medications and therefore lacked the ability and confidence to provide information about medications (Palagyi et al. 2016). Most residents and family caregivers felt emergency department healthcare providers made minimal effort to communicate or engage with them, citing factors such as heavy workloads, understaffing and the prioritisation of other patients (Arendts et al. 2015; Robinson et al. 2012; Sawan et al. 2021). Consequently, many residents and families conveyed feelings of being ‘ignored’ or ‘forgotten’ (Arendts et al. 2015).

Communication challenges persisted upon admission to hospital wards where healthcare providers primarily gave information to residents and family caregivers. However, there were inconsistencies in the manner and quantity of information received. In an observation study, it was revealed that some physicians discussed medication treatments and planning with residents at the bedside, while others, standing at the end of the bed, communicated solely with other healthcare team members, neglecting the resident (McCloskey 2011). Some residents reported receiving minimal information, while others felt overwhelmed by information received from multiple providers, including nurses, pharmacists and specialist physicians (Deeks et al. 2016; Palagyi et al. 2016; Sawan et al. 2021; Toles et al. 2012). Standard hospital practices omitted routinely inviting family members to attend ward rounds, thus excluding them from medication and care plan discussions (Abrahamson et al. 2016; Arendts et al. 2015; Sawan et al. 2021). Family members reported having to initiate discussions with healthcare providers (Abrahamson et al. 2016; Sawan et al. 2021), but often faced limited responsiveness as exemplified in the following statement:

[The hospital] weren't giving her [the resident] the meds that they were giving her at the nursing home…and…she was getting violent…it took 4 days to get the medication she was on in the nursing home. (Abrahamson et al. 2016)

When medication information was provided, it was mostly conveyed verbally and described by residents and family caregivers as heavily jargonised, lacked personalisation and primarily focused on scheduled tests and planning. Consequently, residents and their family caregivers frequently experienced challenges in understanding the provided information, with some being uncertain or unaware of the reasons behind medication changes (Canada et al. 2020; Dyrstad, Laugaland, and Storm 2015; Sawan et al. 2021). During hospitalisation, attempts to involve resident and family caregiver in medication‐related discussions and shared decision‐making were limited or absent, as evidenced by a study showing that 30% of hospitalised residents had no discussions with physicians about their care, and only 33% had engaged in conversations with nursing staff (Toles et al. 2012). Another study found that only 21% of residents had discussed medication changes with hospital healthcare providers (Canada et al. 2020).

#### 4.1.2 Medication Information Exchange at Discharge Experienced by Residents and Families

Nine studies examined discharge communication between residents, families and healthcare professionals during hospital discharge (Abrahamson et al. 2016; Canada et al. 2020; Deeks et al. 2016; Dyrstad, Laugaland, and Storm 2015; McCloskey 2011; Palagyi et al. 2016; Robinson et al. 2012; Sawan et al. 2021; Toles et al. 2012).

Upon hospital discharge, residents and their family caregivers described receiving generic written medication information, such as discharge summaries, without sufficient verbal explanations or counselling. Most felt the volume of information overwhelming, leading to challenges in understanding and remembering it, as expressed by one family caregiver:

The sheer volume of information was very confusing…it all came at discharge. It was too much information at that point in time… (Sawan et al. 2021)

Consequently, many experienced anxiety and confusion about post‐discharge medication management. Family carers of cognitively impaired residents particularly reported deficient communication regarding medications affecting cognition, which prompted many to independently seek information from various sources like the internet, support organisations, pharmacists, primary care physicians and aged care facility staff (Sawan et al. 2021). Most residents regarded the presence of family caregivers during discharge as highly important to support their comprehension of the discharge processes. However, insufficient discharge notice often prevented the presence of caregivers due to competing priorities such as work commitments. Family caregivers were seldom invited to discharge planning meetings with hospital healthcare teams, restricting opportunities to discuss medication management concerns or raise questions (Dyrstad, Laugaland, and Storm 2015; Sawan et al. 2021). Nevertheless, family carers receiving telephone calls from nursing staff regarding ward round discussions reported feeling involved in the discharge process despite their absence (Sawan et al. 2021).

Moreover, the lack of advanced warning about discharge hindered residents and family caregivers from addressing concerns or engaging with healthcare providers about medications and other healthcare matters (Sawan et al. 2021). Although some residents preferred an early discharge, most residents felt pressured to leave hospital to accommodate incoming patients (Canada et al. 2020), often believing their discharge was premature (Dyrstad, Laugaland, and Storm 2015). However, negotiating delayed discharges after expressing their concerns about their readiness to leave the hospital with healthcare providers led to satisfaction among some residents (Dyrstad, Laugaland, and Storm 2015), although this occurred rarely. Some residents and family caregivers without discharge information believed this was because the resident lived in a care facility (Deeks et al. 2016; Sawan et al. 2021). As a consequence, their discharge information needs were overlooked by hospital healthcare professionals who directed all communication back to the care facility. Positive discharge experiences occurred when families received medication lists that provided clear details and instructions or engaged in telephone discussions with healthcare professionals about discharge medication management (Sawan et al. 2021).

Communication challenges between healthcare providers, residents and family caregivers also had an impact on obtaining post‐discharge medications, especially as hospitals typically provided residents with only 3 days' worth at discharge (Deeks et al. 2016; Dyrstad, Laugaland, and Storm 2015; Robinson et al. 2012; Sawan et al. 2021). Family caregivers in particular described the challenges of obtaining refill prescriptions from primary care physicians and community pharmacies especially when discharges occurred during non‐business hours or weekends (Deeks et al. 2016; Sawan et al. 2021), when access to services was limited. Family carers, particularly those caring for residents with cognitive issues, spoke of the pressure they felt to ensure timely medication administration, as illustrated in the statement:

For somebody with dementia and they (the hospital) discharge…on a Friday and the medication takes you to Monday morning. That's not good because you can't get the doctor, I have a lot of trouble getting him anywhere, you know. (Sawan et al. 2021)

Family caregivers spoke of often delaying timely appointments with primary care physicians to obtain prescription refills was often delayed due to residents' compromised health and family caregivers' fatigue stemming from the hospitalisation experience or work commitments (Sawan et al. 2021). In addition, ineffective communication between hospital and primary care physicians often left hospital physicians unaware of the residents' hospitalisation and without timely access to discharge medication information for residents' scheduled appointments. Some family caregivers alleviated the burden by requesting extra discharge medications from hospital staff (Sawan et al. 2021), although this occurred on rare occasions. Other caregivers reported feeling less pressure when hospital pharmacists communicated directly with community pharmacists regarding medication changes, allowing adjustments to be made directly to dose administration aids. However, routine communication between these two pharmacy services did not often occur (Sawan et al. 2021).

#### 4.1.3 Effects of Information Exchange Among Healthcare Staff on Residents and Their Caregivers

All 12 included studies examined issues regarding the exchange of medication information between healthcare professionals during transitions of care.

Residents and family caregivers reported inadequate communication between healthcare providers working within and between healthcare settings during transitions of care. Ineffective information exchange, combined with siloed electronic patient management systems, was identified as a factor amplifying responsibility and pressure on residents and family caregivers (Abrahamson et al. 2016; McCloskey 2011). Often, they found themselves having to address the challenges arising from these deficient communication processes. Residents and family caregivers expected seamless communication between transition settings but were aware that this rarely occurred (Abrahamson et al. 2016; Arendts et al. 2015; McCloskey 2011). In one example, a resident shared an experience of sudden medication cessation without tapering in the hospital, which was not communicated to the aged care home nursing staff:

They [the hospital] took me off… Cymbalta…you're not supposed to just stop taking it. You're supposed to wean off of it. They didn't. But I got that back yesterday. (Canada et al. 2020)

Some family members reported how inconsistencies in the hospital discharge summary led to medication administration errors:

While he [the resident] was in the hospital…the geriatrician…said we are going to stop the oxycodone… On the discharge summary, which we got two weeks later, they actually discharged him with oxycodone. (Sawan et al. 2022)

In one study (McCloskey 2011), hospital physicians were observed communicating abbreviated discharge medication instructions, such as ‘there are no new medications’ or ‘prescriptions in the envelope’, to paramedics prior to convey to aged care home nursing staff. However, due to other ambulance requests, paramedics often lacked time to communicate this information, elevating the risk of medication discrepancies between hospital and aged care home (McCloskey 2011). Despite routine use, standardised patient information forms containing medication details did not enhance medication information exchange among healthcare providers in different settings (McCloskey 2011). Observations revealed instances where the forms were not received or were disregarded or misinterpreted by the receiving healthcare providers (McCloskey 2011). In one study, a scenario was described where an emergency department physician received a resident with no accompanying information, was unable to determine why the resident was in the emergency department and therefore transferred the resident back to the aged care home untreated (Deeks et al. 2016). A number of practical challenges regarding the use of transfer forms were cited across studies. For example, the information documented on these forms often did not match the receiver's requirements (McCloskey 2011).

### 4.2 Resident and Family Factors Influencing Medication Communication Engagement

Several factors related to residents and their family caregivers were found to influence medication communication with healthcare providers across all 12 studies. These factors included resident and family caregiver knowledge about medications, their preferences for involvement in medication‐related decision‐making and family caregiver advocacy.

#### 4.2.1 Knowledge About Prescribed Medications

Nine studies included in this review examined the impact that resident and family member factors and preferences influenced communication about medication management during transitional care (Canada et al. 2020; Deeks et al. 2016; Dyrstad, Laugaland, and Storm 2015; Gjerberg et al. 2015; McCloskey 2011; Palagyi et al. 2016; Sawan et al. 2021; Sawan et al. 2022; Toles et al. 2012).

While recalling specific medication details proved challenging for most residents, they could generally provide information about the number, appearance, taste, negative side effects and routines. However, a lack of specific medication knowledge, such as indications, actions and potential adverse events, was evident among most residents and their family caregivers (Palagyi et al. 2016). This knowledge gap seemed to contribute to a general apathy towards medications, particularly polypharmacy and potentially inappropriate medications. Even when the daily medication count was considered excessive, neither residents nor families were motivated to seek change (Palagyi et al. 2016). A resident expressed this acceptance, stating:

I've got a lot [of medications]…Love it be less, but if I need that many, I need that many and I've got to take them and bear with it. (Sawan et al. 2022)

For residents, this indifference appeared to be related to their poor levels of health or their complete trust in the care and decision‐making of healthcare providers, especially physicians (Palagyi et al. 2016). Some residents lacked awareness of their rights to inquire about their medications (Sawan et al. 2022). For family caregivers, the reasons for apathy were less unclear, but they too often placed great confidence in the medication management of healthcare providers. As a result, these residents and family caregivers often delegated all aspects of medication management to healthcare providers.

Residents' beliefs about medications also impacted their involvement in discussions with healthcare providers. Some were unmotivated to participate in decision‐making because they believed their medications were beneficial and not a safety risk (Dyrstad, Laugaland, and Storm 2015; Gjerberg et al. 2015; Sawan et al. 2022). Others deemed that medication‐related discussions were unnecessary because of the absence of side effects or because residents had been taking medications for a long period of time:

I don't need to discuss medicines with my doctor, cause I've been taking them virtually all my life. (Sawan et al. 2022)

Some residents also expressed fear over potential consequences if medications were reduced or ceased, such as the return of symptoms or even death:

That's the only way I'm still walking on two legs. If I didn't have it [medication] I'd be probably already six foot under the ground. (Palagyi et al. 2016)

Others stated that they were aware of other residents who had trialled cessation and were unsuccessful (Sawan et al. 2022).

#### 4.2.2 Preferences for Involvement in Decision‐Making

Seven studies investigating preferences for involvement in decision‐making regarding medication management found a diverse spectrum of inclinations, ranging from minimal to high engagement (Abrahamson et al. 2016; Dyrstad, Laugaland, and Storm 2015; Gjerberg et al. 2015; Palagyi et al. 2016; Sawan et al. 2021; Sawan et al. 2022; Toles et al. 2012). At one end of the spectrum, residents and family caregivers expressed a preference for little or no involvement, entrusting healthcare providers with sole responsibility for medication‐related decisions. For some, this preference stemmed from trust in the safety and benefits of the prescribed medications or a desire to delegate the responsibility to healthcare providers (Sawan et al. 2022). Some residents and family caregivers believed it was not their role to participate in medication decision‐making as they perceived healthcare providers were the authority figures in medical matters and believed that decisions about medications should be the responsibility of trained professionals (Gjerberg et al. 2015; Palagyi et al. 2016; Sawan et al. 2022). Others felt incapable of active involvement due to a limited understanding of the residents' medications, medical conditions and treatments (Palagyi et al. 2016; Sawan et al. 2022). For example, most family caregivers expressed a willingness to participate in decision‐making, but some hesitated due to the perceived burden of making final decisions as expressed by one family caregiver:

… I want to be part of the conversation, I want to participate in the discussion of medication and such, but specifically saying yes or no, I feel like that is too much responsibility for me. (Gjerberg et al. 2015)

Conversely, other residents and family caregivers exhibited a preference for participation or full decision‐making authority (Dyrstad, Laugaland, and Storm 2015; Gjerberg et al. 2015; Palagyi et al. 2016; Sawan et al. 2022; Toles et al. 2012). These individuals wanted to collaborate with healthcare providers to make informed decisions aligned with their preferences and values. In one study (Sawan et al. 2022), certain residents, including those with limited English proficiency, low health literacy, and compromised health, asserted their right to engage in medication discussions. For these residents, self‐advocacy played a crucial role in maintaining control over their health and well‐being. Those wanting involvement in decision‐making faced obstacles to engaging in discussions with healthcare providers. Challenges included limited opportunities provided by healthcare providers to engage due to busy workloads, the prioritisation of other patients, as well as pressure to conserve resources, such as hospital bed availability and adherence to a medicalised model of care focused on treating conditions rather than individuals (Sawan et al. 2021). Furthermore, the absence of structured communication routines across settings often led to residents and family caregivers being sidelined from the decision‐making process (Sawan et al. 2021).

#### 4.2.3 Family Caregiver Advocacy

Seven studies addressed the advocacy of families during transitional care (Abrahamson et al. 2016; Arendts et al. 2015; Deeks et al. 2016; Dyrstad, Laugaland, and Storm 2015; Gjerberg et al. 2015; Robinson et al. 2012; Sawan et al. 2021).

Many family caregivers described themselves as resident advocates during transitions of care For many, this assumed role stemmed from their concerns about perceived deficiencies in communication between acute and community settings and between healthcare providers and residents. Family carers felt the need to ‘keep an eye on things’ and be an advocate, particularly in the hospital setting, because residents would not, or could not, speak up for themselves, as many residents were in poor mental and physical health. Advocacy involved monitoring the care received, providing feedback to staff and filling information gaps. In some cases, this entailed speaking up and demanding action from healthcare providers. Many family members perceived themselves as important advocates because of the extensive knowledge of their resident and therefore are well placed to notice changes in health status during transitions of care (Robinson et al. 2012). Family caregivers also provided reassurance, security and clarification of medication information to residents (Robinson et al. 2012; Sawan et al. 2021), which was highly valued by residents:

My son received the necessary information and explained the treatment plan to me. (Dyrstad, Laugaland, and Storm 2015)

It is good having my daughter present when information is given; it makes me feel safe. (Dyrstad, Laugaland, and Storm 2015)

Family carers also assisted residents in navigating the healthcare system, which was often out of necessity due to limited communication with healthcare professionals. This included asking questions about and coordinating follow‐up medications, transportation and collecting medications (Sawan et al. 2021). However, many family members faced challenges in ensuring their involvement and advocacy due to being ignored by healthcare practitioners. As a result, some family members admitted that they gave up, stating:

…I gave up and just thought what's the point. You know, I've got to there's no point, they're [hospital healthcare professionals] just going to do what they want. (Sawan et al. 2021)

## 5 Discussion

This review showed that health systems lacked a structure conducive to supporting effective communication and collaborative resident and family‐centred practices during transitions of care. Communication primarily consisted of one‐way interactions, with residents and family caregivers receiving or providing medication information to healthcare providers. Studies included in the review also revealed minimal evidence of active engagement or shared decision‐making by healthcare providers with residents and family carers and a lack of family caregiver involvement in medication management communication. Residents and their family caregivers had individual needs and perspectives regarding medication communication, but these were not well addressed during transitions of care. Several factors that appeared to influence communication dynamics included time constraints and heavy workloads among healthcare providers, residents, and family caregiver preferences for involvement in decision‐making and residents' beliefs about medications.

Throughout transitions of care, the communication experiences concerning medication management for residents and family caregivers primarily revolved around providing or receiving information, a trend consistent with previous research in aged care home settings (Manias et al. 2021) and during transitions of care for older patients and their families (Belcher et al. 2006; Manias et al. 2019; Ozavci et al. 2021; Tobiano et al. 2021). This provision of information indicates that communication tends to focus on exchanging medication‐related details rather than promoting active engagement or shared decision‐making among older patients, family caregivers and healthcare providers. Furthermore, regardless of the specific context or phase of the transition journey, the communication patterns of healthcare providers seem to remain relatively unchanged. Only one study documented occasions where hospital physicians actively engaged with residents, indicating that this was an infrequent scenario (McCloskey 2011).

In healthcare, involving residents and family caregivers is integral to person‐centred care, which emphasises meeting patients' and family carers' needs through enhanced communication (Australian Commission on Safety and Quality in Health Care 2011; Giusti et al. 2022). However, our finding that person‐centred care was rarely experienced by residents or their caregivers highlights significant challenges in healthcare delivery during transitions of care. For example, inadequate communication inhibited both groups from fully understanding medication plans, treatment options and potential risks. Without comprehensive information, they often struggled to make informed decisions, leading to feelings of uncertainty and anxiety. Many residents and family caregivers also felt disempowered in decision‐making processes, and this lack of involvement led to decreased satisfaction with transitional care experiences and perceptions of being overlooked or undervalued by healthcare providers. This was evidenced by the limited knowledge of medications among residents and families, which led to a reluctance among some in discussing them with healthcare providers.

Our review also revealed a consistent absence of family caregivers in medication communication with healthcare providers during transitions of care. While prior research has noted this in studies on caregiver involvement for older people living in their own homes (Bragstad, Kirkevold, and Foss 2014; Bull, Hansen, and Gross 2000; Digby, Moss, and Bloomer 2011; Jeffs et al. 2017), a gap exists in understanding the involvement of caregivers of residents during transitions of care. Family caregiver involvement is crucial during these transitions, given the communication hurdles that residents frequently encounter, stemming from factors like cognitive impairment, poor physical health and confusion. However, family caregivers also encounter obstacles in communicating with healthcare providers, including role ambiguity, lack of opportunity for engagement and exclusion from shared decision‐making. Despite many family caregivers considering themselves authorities on residents' needs and preferences, they often experienced frustration and role confusion when their knowledge was overlooked by healthcare providers and instead were asked to provide only specific details related to the residents' diagnoses and treatments.

Although studies show that healthcare providers acknowledge the importance of caregivers during care transition (Jeffs et al. 2017), our review exposes a stark underutilisation of family caregivers as valuable sources of support and information. This oversight may contribute to the generalised lack of confidence observed among many family caregivers in the abilities of healthcare providers to communicate effectively and provide adequate care for residents. As evidenced by their belief that they must advocate for residents' medication and other care needs, as well as navigate the complexities of the healthcare system, family caregivers felt compelled to take on additional responsibilities. This finding is consistent with prior research indicating that caregivers perceive the need to advocate for their elderly relatives during transitions of care for more information and involvement in care planning in order to be prepared (Bragstad, Kirkevold, and Foss 2014; Byrne, Orange, and Ward‐Griffin 2011; Hvalvik and Reierson 2015; Jeffs et al. 2017). Additionally, caregivers often find themselves having to navigate the healthcare system and service delivery for older patients due to lack of information and support provided by healthcare providers (Bragstad, Kirkevold, and Foss 2014; Byrne, Orange, and Ward‐Griffin 2011; Hvalvik and Reierson 2015; Levine et al. 2010). This highlights the role of family caregivers as intermediaries between residents and healthcare services (Sherman 2019). In addition, our study identified that healthcare providers did not initiate discussions with caregivers regarding their preferences for involvement in medication management or other aspects of care, leading family caregivers no option but to take a proactive stance. This aligns with other studies showing caregivers want greater involvement and receive the same information provided to their elderly patients (Bragstad, Kirkevold, and Foss 2014; Hvalvik and Reierson 2015). Further research is needed to understand the preferences of caregiver roles in medication management during transitions and use this information to explore strategies to engage and support caregivers in medication communication.

Our review highlights a relatively unexplored area regarding how residents' beliefs about medications may influence communication with healthcare providers about medication management. Previous research has predominantly focused on the medication beliefs of older patients living independently, examining their impact on medication adherence (Clyne et al. 2017; Gaffney and Hamiduzzaman 2022; Hong 2019) and their interactions with healthcare providers (Ozavci et al. 2022). It has been reported in studies that patient‐provider communication significantly influences medication beliefs, as it fosters understanding and trust regarding medications (Young et al. 2017). Older patients' compliance with medication regimens often hinges on their perceptions of medication necessity and benefit (Allen LaPointe et al. 2011), alongside a reluctance to question healthcare providers' decision‐making (Ozavci et al. 2022) and that older patients may assume effectiveness and benefit because the medications are prescribed (Volume, Burback, and Farris 1999). In addition, throughout their life, older residents will have had frequent encounters with medication use and, thus, will have formed firm medication beliefs. Our study also revealed that many residents prioritised belief in the necessity of their medications and trust in physician decision‐making over concerns about their medications with healthcare providers. This could lead to a limited awareness of medication risks and benefits, acceptance of inappropriate medications and reluctance to address concerns or side effects, potentially resulting in adverse drug reactions. Previous research has shown that trust in healthcare providers may foster a willingness to accept medications, including polypharmacy, and their willingness to consider deprescribing (Anderson et al. 2014; Anthierens et al. 2010; Cullinan et al. 2014; Schuling et al. 2012). Therefore, it is important for healthcare providers to understand residents' medication beliefs to facilitate effective, person‐centred communication with residents and their caregivers to improve medication management outcomes.

## 6 Limitations and Strength of the Work

The systematic review has several limitations. Studies not conducted in English were excluded, potentially narrowing the scope of the review's findings. Only one study provided participant information beyond age and gender, making it challenging to explore the influence of other characteristics, such as ethnicity or primary language, on communication with healthcare providers. The review's strengths lie in synthesising data from studies conducted in multiple western countries which enhances the transferability of findings to similar contexts. Furthermore, the studies included a mixture of residents with and without cognitive impairments, which increases the potential transferability of findings to aged care contexts in western countries. Lastly, the use of interviews, focus groups and observations across the 12 studies included in this review allowed for triangulation of data, bolstering the credibility and reliability of the findings. Combining these methods allowed us to capture a holistic understanding of participants' experiences and behaviours in different contexts, contributing to the rigour and depth of the topic under review.

## 7 Future Research

Future research initiatives hold significant promise due to the limited exploration of residents' and family caregivers' involvement in medication management during transitions of care. Utilising qualitative methods such as interviews, observations and focus groups in subsequent studies would elucidate the communication dynamics among residents, caregivers and healthcare providers throughout the transition process. Understanding their perceptions, roles and communication needs can inform innovative strategies to enhance medication management practices benefiting all stakeholders. Co‐design methodologies with stakeholders can refine these strategies, ensuring practicality and viability across the transitional care continuum. For instance, research could involve developing educational materials tailored to residents and family caregivers for each transition phase. Both groups require tailored resources to meet their information needs as they transition across different health settings. Future investigation into the medication management experiences and needs of specific resident groups, like those with cognitive impairment and language barriers, alongside their family caregivers, hold immense value. Observation inquiries could delve into how factors, such as beliefs and values, language and communication, health literacy, social support networks and cultural norms, influence their medication management communication during transitions of care (Manias 2010). Furthermore, these studies could assess the impact of health system elements like environment, healthcare provider availability and geriatric skills. Extending these investigations to healthcare providers can help identify gaps in communication practices and enhance outcomes for residents, family caregivers and healthcare providers. Tailored interventions can then be developed to ensure medication management meets the diverse needs of vulnerable resident populations and considers various influencing factors, ultimately promoting more effect and patient‐centred care.

## 8 Implications for Practice

Healthcare providers involved in transitions of care, medication management and resident care should use medication management as an opportunity to educate residents and their family caregivers about medication changes. They should actively engage residents and family caregivers during key communication processes like ward rounds, handovers and informal discussions, addressing concerns and involving them in decision‐making. Healthcare providers should serve as advocates during these interactions, ensuring residents and caregivers are included in medication decision‐making.

Healthcare providers can educate residents and family caregivers using various communication methods and tools such as teach back methods, chunk and check, use of patient care boards and illustrations, bedside handovers, multidisciplinary huddles, and ward rounds (Roodbeen et al. 2020). Education resources could help improve the communication and shared decision‐making process between providers, residents, and family caregivers. Various formats, including face‐to‐face, written and visual materials, are available online (Roodbeen et al. 2020). Hospital pharmacists have the opportunity to expand their role in educating residents and family caregivers about medications, especially during discharge, when the process can be often overwhelming for them.

Considering the perspectives of residents and their family caregivers is crucial for addressing their concerns and promoting patient‐centred care (McDerby et al. 2020). Effective medication communication requires a multidisciplinary team approach, leading to improved patient satisfaction and reduced hospital readmissions (Ozavci et al. 2021). Multidisciplinary teams facilitate communication among healthcare providers, patients and families, promoting shared decision‐making and ensuring patients are well‐informed about their medications. Tailoring care plans to individual needs enhances patient satisfaction and adherence. Patient‐centred medication management, best achieved through multidisciplinary planning and practice (Liu, Gerdtz, and Manias 2016), should be provided to residents and their caregivers at each transition point. In addition, this review underscores that residents' and family caregivers' participation in medication communication during care transitions is shaped by various factors. These include their knowledge and beliefs about medications, as well as perceptions of the transition environment (e.g., time constraints on healthcare providers, hurried discharges). Healthcare providers must recognise these influences on communication and respond by actively listening to residents' and family caregivers' concerns, prompting them to ask questions for clarity on medication management plans and fostering trusting relationships.

The results of the qualitative meta‐synthesis provide important recommendations for enhancing communication and engagement. Healthcare systems often do not prioritise the active involvement of family caregivers or residents in communication during transitions of care. Nonetheless, strategies can be implemented to enhance engagement of residents and caregivers in medication management communication in the near term. Settings such as hospitals and aged care homes can significantly boost family caregiver involvement in medication decision‐making discussions by adopting video conferencing technology. Through this, caregivers can actively engage in crucial healthcare conversations regardless of location, offering a quick and accessible solution for these settings. The experience gained from the COVID‐19 pandemic, where reducing in‐person contacts prompted the deployment of video consultations, means many institutions likely have the resources available for this. Integrating video conferencing into daily routines allows healthcare teams to seamlessly include caregivers in pivotal moments like bedside ward rounds in hospitals, while in aged care homes, virtual meetings enable caregivers to discuss medication regimens and treatment adjustments with visiting physicians or nursing staff. Additionally, hospitals and aged care homes could organise virtual medication education sessions where healthcare providers provide information on medications, potential side effects, and administration techniques to residents living in aged care homes and family caregivers. The presence of family caregivers during these discussions also benefits residents, as they feel supported and empowered by their advocacy, enhancing their sense of agency in care decision‐making.

In addition, aged care homes could enhance their medication management education strategies by expanding the role of medicines governance groups, such as Medication Advisory Committees, typically comprised of physicians, registered nurses, pharmacists and consumers (Australian Government Department of Health and Aged Care 2022). These groups are responsible for medication policy development, risk management advice, identifying educational needs and monitoring effectiveness to improve medication management quality (Australian Government Department of Health and Aged Care 2022).

To better engage with residents and family caregivers, these groups could create tailored educational materials, organise workshops covering medication management topics and ensure access to relevant resources like medication information leaflets and user‐friendly online tools, including free apps like Heathdirect, MedicineWise and MedAdvisor, aimed at improving communication with healthcare providers and medication management. Aged care homes could swiftly adopt these strategies, which would be pivotal in empowering residents and family caregivers with the requisite knowledge and skills to adeptly manage medications and communicate confidently with healthcare providers.

Healthcare providers must communicate about medications with residents in clear, understandable terms to ensure comprehension and encourage their active involvement in healthcare decisions. This communication should occur proactively before any medication changes, especially during transitions between care settings. Engaging residents in discussions about their medications empowers them to participate in decision‐making processes regarding their treatment plans. Various communication methods, including clear explanations, visual aids and tools like SBAR (Situation, Background, Assessment, Recommendation) (American Society for Quality 2024), facilitate effective communication between healthcare providers and residents during handovers, consultations and other interactions. Additionally, providing residents with a straightforward medication list detailing their prescriptions, dosages and administration instructions enhances their understanding and management of their medication regimen.

## 9 Conclusion

Our review underscores several critical findings regarding medication management communication during transitions of care. Firstly, communication predominantly focuses on exchanging medication information rather than fostering active engagement or shared decision‐making among older patients, family caregivers and healthcare providers. Secondly, healthcare providers' communication patterns remain relatively unchanged across different transition phases, with limited instances of active engagement with residents. Moreover, our study highlights a stark underutilisation of family caregivers as valuable sources of support and information during care transitions, despite their perceived roles as advocates for residents' needs. Additionally, residents' beliefs about medications strongly influence their communication with healthcare providers, with many prioritising trust in physicians' decisions over concerns about their medications. Addressing these challenges requires tailored interventions to enhance communication practices and promote person‐centred care throughout transitions of care, ultimately improving medication management outcomes for residents and their caregivers.

## Author Contributions

Conceptualisation of the systematic review was conducted by A.D. and E.M. Data curation was conducted by all authors (i.e., A.D., S.G. and E.M. were responsible for data collection and screening). Formal analysis was undertaken by A.D. and E.M. A.D. and E.M. conducted analysis and interpretation of data. A.D. was responsible for writing the original draft. S.G. and E.M. were responsible for review and editing of the original draft. All authors have agreed on the final version of the review.

## Ethics Statement

The authors have nothing to report.

## Conflicts of Interest

The authors declare no conflicts of interest.

## Supporting information


Appendix S1

Appendix S2

Appendix S3

Appendix S4

## Data Availability

Data sharing is not applicable to this article as no new data were created or analyzed in this study. A registered repository is not required for the data presented in this qualitative meta‐synthesis manuscript. The data utilized in the meta‐synthesis originates from accessible published manuscripts, eliminating the need for a separate repository. Detailed information extracted from each included manuscript is meticulously aggregated within the meta‐synthesis, including in the Section 3, alongside Figure 1, Tables 1 and 2, and Appendices [Link], [Link]. Since primary data sources are openly accessible through published literature, there is no requirement for a separate repository, and thus, the data does not necessitate a Persistent Identifier or Digital Object Identifier. This qualitative meta‐synthesis relies on synthesizing and analyzing existing published literature rather than generating new empirical data, making a registered repository redundant. The data used in this meta‐synthesis is sourced from publicly available peer‐reviewed journals, obviating the necessity for a registered repository, as the study relies on secondary sources rather than original data collection.
