# Supplementary material for: Experiences and Perceptions of Medication Management Communication During Transitions of Care for Residents in Aged Care Homes and Their Caregivers: A Qualitative Meta‐Synthesis
Source: J Clin Nurs. 2024 Oct 6;34(4):1432–51. doi: 10.1111/jocn.17438 (PMC11933520; doi:10.1111/jocn.17438)
Supplement: Supplementary file 2 — Appendix S2 [file JOCN-34-1432-s002.docx]

**Supplementary File 2: Database search terms and results**

| **Database** | **Search strategy** | **Search results based on the inclusion criteria (*n)*** |
| --- | --- | --- |
| Medline Ovid R | aged/ OR aged.ti.ab. OR fraily elderly/ OR elderly.ti.ab. OR Aged, 80 and over/ OR (older adj (person*)).ti.ab. OR Caregivers/ or caregiver*.ti.ab. OR (family adj(member* or caregiver*)).ti.ab. AND communication.ti.ab. OR Polypharmacy/ OR Medication errors/ OR Desprescriptions/ OR (medical adj(record* or information or documentation or communication)).ti.ab. OR Medication Therapy Management/ OR (medication adj2(error* or use or usaage or utili#ation or management or reconciliation or evaluation or review or communication or information or adherence)).ti.ab. or Drug Utilization Review/ AND Transitional Care/ OR transitions of care.ti.ab. OR (patient* adj(transport or transfer*).ti.ab. OR (transfer* adj5(hospital or nursing home* or emergency)).ti.ab. OR (transfer* adj2 older person*).ti.ab. | 449 |
| Embase | Aged/ OR aged.ti.ab. OR fraily elderly/ OR elderly.ti.ab. OR Very Elderly/ OR (older adj (people or person*)).ti.ab. OR Nursing Home Patient/ OR (nursing home adj2(resident* or patient*)).ti.ab OR Caregiver/ or caregiver*.ti.ab OR (family adj(member* or caregiver*)).ti.ab. AND communication.ti.ab. OR Polypharmacy/ OR Prescribing error/ OR Deprescription/ OR (medical adj(redord* or information or documentation or communication)).ti.ab. OR Medication Therapy Management/ OR (medication adj2(error* or use or usage or usitli#ation or management or reconciliation or evaluation or review or communication or information or adherence)).ti.ab. AND Drug Utilization Review/ AND Transitional Care/ OR transitions of care.ti.ab. OR Patient transport/ OR (patient* adj(transport or transfer*)).ti.ab OR (transfer* adj5(hospital or nursing home* or emergency)).ti.ab. OR (transfer* adj2 older person*)).ti.ab OR (resident* adj2(transfer* or transition*)).ti.ab. AND Nursing home/ OR (nursing adj home*).ti.ab. OR Long term care/ OR ((long term or residential) adj2 care).ti.ab. OR Home for the aged/ OR Emergency care/ OR Hospital emergency service/ OR (emergency adj2(medicine or service* or ward*)).ti.ab. OR Hospital discharge/ OR ((patient or hospital) adj2 discharge).ti.ab. OR discharge planning.ti.ab. | 608 |
| PsychINFO | Aged/ OR aged.ti.ab. OR elderly.ti.ab. OR (older adj(people or person*)).ti.ab. OR Caregivers/ OR caregiver*.ti.ab. OR (family adj(member* or caregiver*)).ti.ab AND communication.ti.ab. OR Polypharmacy/ OR (medical adj(record* or information or documentation or communication)).ti.ab. OR (medication adj2(error* or use or usage or utli#ation or management or reconciliation or evaluation or review or communication or information or adherence)).ti.ab. AND transitions of care.ti.ab. or (patient* adj(transport or transfer*)).ti.ab. OR (transfer* adj5 (hospital or nursing home* or emergency)).ti.ab. OR (resident* adj2 (transfer* or transition*)).ti.ab. AND (nursing adj home*).ti.ab. OR Long term care/ OR ((long term or residential) adj2 care).ti.ab. OR (emergency adj2 (medicine or service* or ward*)).ti.ab. OR Hospital discharge/ OR ((patient or hospital) adj2 discharge).ti.ab. OR Discharge planning/ | 72 |
| Scopus | "aged patient" OR "aged people" OR "aged person" OR "elderly" OR "elderly patient" OR "elderly people" OR "frail elderly" OR "older people" OR "older person*" OR "older patient*" OR "nursing home patient*" OR "nursing home resident*" OR "long term care patient" OR "long term care resident*" OR pre/n: "nursing home" PRE/2 resident* OR "caregiver*" OR "care giver*" OR "carer*" OR "family caregiver*" OR "family care giver*" AND "medication communication" OR "polypharmacy" OR "prescri* error*" OR "misprescri*" OR "inappropriate prescri*" OR "deprescri*" OR "de prescri*" OR pre/n: AND medical PRE/1 record* OR pre/n: AND medical PRE/1 documentation OR pre/n: AND medical PRE/1 communication OR "medication therapy management" OR "drug therapy management" OR "medication management" OR "medication reconciliation" OR pre/n: AND medication PRE/2 error* OR pre/n: AND medication PRE/2 us* OR pre/n: AND medication PRE/2 management OR pre/n: AND medication PRE/2 reconciliation OR "drug utilization review" OR "drug usage review" OR "drug utili$ation evaluation" OR "drug utili$ation review" OR "medication review*" OR "medication use review" OR "medication us*" AND "transitional care" OR "transition care" OR "transition* of care" OR "care transition*" OR “patient transport*” or “intrahospital transfer*” or “transport, patient*” or “transportation of patient*” OR Pre/n: patient* Pre/1 transport* OR Pre/n: patient Pre/1 transfer* OR Pre/n: transfer* Pre/5 hospital OR Pre/n: transfer* Pre/5 emergency AND “nursing home*” or “long term care” or “residential aged care” or “aged care home*” or “assisted living facility*” OR Pre/n: “long term” Pre/2 care OR Pre/n: “residential” Pre/2 care OR “geriatric* home*” or “home* for the elderly” or “homes for the aged” or “old age home*” or “old people* home*” OR “emergency care” or “acute care” or “emergency health care” or “emergency medical care” OR “hospital discharge” or “discharge plan*” or “patient discharge” | 307 |
| CINAHL | (MH "Aged") OR (MH "Aged, 80 and Over") OR (MH "Frail Elderly") OR (MH "Nursing Home Patients") OR Care#giver OR “carer*” or “caregiver*” or “care giver*” OR “family care*” or “informal care*” AND (MH "Communication") OR (MH "Polypharmacy") OR (MH "Medication Errors") OR (MH "Deprescribing") OR medical record review or medical information or medical documentation or medical communication OR (MH "Medication Management") OR medication errors OR medication use OR medication usage OR medication reconciliation OR medication review OR medication communication OR medication information OR medication adherence OR (MH "Record Review") AND (MH "Transitional Care") OR transitions of care OR (MH "Transportation of Patients") OR patient transport or patient transfer or patient transition OR (MH "Transfer, Discharge") AND (MH "Nursing Homes") OR (MH "Long Term Care") OR (MH "Residential Facilities") or (MH "Residential Care") OR (MH "Emergency Care") or (MH "Emergency Service") OR (MH "Transfer, Discharge") OR (MH "Discharge Planning") | 364 |
| EmCare | Aged/ OR aged.ti.ab. OR Frail elderly/ OR Elderly.ti.ab. OR Very elderly/ OR (older adj(people or person*)).ti.ab. OR Nursing home patient/ OR (nursing home adj2 (resident* or patient*)).ti,ab. OR Caregiver/ OR caregiver*.ti.ab. OR (family adj (member* or caregiver*)).ti,ab. AND communication.ti.ab. OR Polypharmacy/ OR Prescribing error/ OR Deprescription/ OR (medical adj (record* or information or documentation or communication)).ti,ab. OR medication therapy management/ OR (medication adj2 (error* or "use" or "usage" or "utili#ation" or management or reconciliation or evaluation or review or communication or information or adherence)).ti,ab.OR "drug utilization review"/ AND Transitional care/ OR transitions of care.ti.ab. OR Patient transport/ OR (patient* adj (transport or transfer*)).ti,ab. OR (Transfer* adj5 (hospital or nursing home* or emergency)).ti,ab. OR (Transfer* adj2 older person*).ti,ab. OR (Resident* adj2 (transfer* or transition*)).ti,ab. AND Nursing home/ or (nursing adj home*).ti,ab. OR Long term care/ OR ((long term or residential) adj2 care).ti,ab. OR Home for the aged/ OR Emergency care/ or Hospital emergency service/ OR (emergency adj2 (medicine or service* or ward*)).ti,ab. OR Hospital discharge/ OR ((patient or hospital) adj2 discharge).ti,ab. OR discharge planning.ti,ab. | 300 |
| Web of Science | "aged" or "elder*" OR "nursing home patient" or "nursing home resident" OR "older people" or "older person*" OR "aged care home resident*" or "aged care home patient*" OR "caregiver"* or "care giver" or "carer*" or "family carer*" or "family caregiver*" or "family member" or "family care giver*" or “geriatric patient*” or “older” or “frail*” AND "communication" or “medication communication” or "medication*" or "medication information" or "medication therapy" or "medication review*" or "documentation" or “patient centered care” or “patient centred care” or “decision making” AND "transitional care" or "transitions of care" or "patient transport" or "patient transfer*” or "resident* transfer*" or "resident* transition*" or “handover*” or “patient handover*” AND “nursing home*” or “long term care” or “residential care” or “assisted living” or “aged care home*” or “emergency department*” or “emergenc*” or “hospital*” or “emergency care” or “admission*” or “discharge*” or “discharge plan*” or “patient discharge” or “hospital discharge” | 510 |
