# Supplementary material for: Experiences and Perceptions of Medication Management Communication During Transitions of Care for Residents in Aged Care Homes and Their Caregivers: A Qualitative Meta‐Synthesis
Source: J Clin Nurs. 2024 Oct 6;34(4):1432–51. doi: 10.1111/jocn.17438 (PMC11933520; doi:10.1111/jocn.17438)
Supplement: Supplementary file 3 — Appendix S3 [file JOCN-34-1432-s001.docx]

**Supplementary File 3: Assessment of the qualitative studies: Joanna Briggs Institute (JBI) Critical Appraisal Checklist for Qualitative Research (Lockwood et al 2015)**

| **JBI Question** | **Author, Date** | | | | | | | | | | | |
| --- | --- | --- | --- | --- | --- | --- | --- | --- | --- | --- | --- | --- |
|  | **Abrahamson (2016)** | **Arendts  (2015)** | **Canada (2020)** | **Deeks (2016)** | **Dyrstad (2014)** | **Gjerberg (2015)** | **McCloskey (2011)** | **Palagyi (2016)** | **Robinson (2012)** | **Sawan  (2021)** | **Sawan  (2022)** | **Toles (2012)** |
| **1.Is there congruity (“fit”) between the stated philosophical perspective and the research methodology?** | Yes | Yes | Yes | Yes | Yes | Yes | Yes | Yes | Yes | Yes | Yes | Yes |
| **2.Is there congruity between the research methodology and the research question or objectives?** | Yes | Yes | Yes | Yes | Yes | Yes | Yes | Yes | Yes | Yes | Yes | Yes |
| **3.Is there congruity between the research methodology and the methods used to collect data?** | Yes | Yes | Yes | Yes | Yes | Yes | Yes | Yes | Yes | Yes | Yes | Yes |
| **4.Is there congruity between the research methodology and the representation and analysis of data?** | Yes | Yes | Yes | Yes | Yes | Yes | Yes | Yes | Yes | Yes | Yes | Yes |
| **5.Is there congruity between the research methodology and the interpretation of results?** | Yes | Yes | Yes | Yes | Yes | Yes | Yes | Yes | Yes | Yes | Yes | Yes |
| **6.Is there a statement locating the researcher culturally or theoretically?** | No | No | No | No | No | No | No | No | No | No | No | No |
| **7.Is the influence of the researcher on the research, and vice- versa, addressed?** | No | No | No | No | No | No | No | No | No | Yes | No | No |
| **8.Are participants, and their voices, adequately represented?** | Yes | Yes | Yes | Yes | Yes | Yes | Yes | Yes | Yes | Yes | Yes | Yes |
| **9.Is the research ethical according to current criteria or, for recent studies, and is there evidence of ethical approval by an appropriate body?** | Yes | Yes | Yes | Yes | Yes | Yes | Yes | Yes | Yes | Yes | Yes | Yes |
| **10.Do the conclusions drawn in the research report flow from the analysis, or interpretation, of the data?** | Yes | Yes | Yes | Yes | Yes | Yes | Yes | Yes | Yes | Yes | Yes | Yes |
