# Supplementary material for: Experiences and Perceptions of Medication Management Communication During Transitions of Care for Residents in Aged Care Homes and Their Caregivers: A Qualitative Meta‐Synthesis
Source: J Clin Nurs. 2024 Oct 6;34(4):1432–51. doi: 10.1111/jocn.17438 (PMC11933520; doi:10.1111/jocn.17438)
Supplement: Supplementary file 4 — Appendix S4 [file JOCN-34-1432-s004.docx]

**Supplementary File 4: Examples of Consolidated Criteria for Reporting Qualitative Studies (COREQ): 32-item checklist for**

**Example 1:** Abrahamson et al (2016) “The experiences of family members in the nursing home to hospital transfer decision”

| **No** | **Item** | **Guide questions/description** | **Response/Page** |
| --- | --- | --- | --- |
| **Domain 1: Research team and reflexivity** | | |  |
| Personal Characteristics | | |  |
| 1. | Interviewer/facilitator | Which author/s conducted the interview or focus group? | √ Authors who conducted the interviewers are described in the method (Page 2) |
| 2. | Credentials | What were the researcher's credentials? *E.g. PhD, MD* | X Researchers’ credentials are not described |
| 3. | Occupation | What was their occupation at the time of the study? | X Researchers’ occupations are not described |
| 4. | Gender | Was the researcher male or female? | √ The gender of the authors can be assumed from their Christian names (ie 4 females and 1 male) (Page 1) |
| 5. | Experience and training | What experience or training did the researcher have? | X Researchers’ experience and training is not described. However, reflexivity was reported and facilitated by on-going dialogues with the authors throughout data collection and analysis. |
| Relationship with participants | | |  |
| 6. | Relationship established | Was a relationship established prior to study commencement? | X There is no evidence of an established relationship with participants although the project was part of a larger, 4-year project, so it is possible that the authors/interviewers had established some prior relationship with the family members. |
| 7. | Participant knowledge of the interviewer | What did the participants know about the researcher? e*.g. personal goals, reasons for doing the research* | √ Authors indicate that the project involved a registered nurse (RN) in each facility to lead the delivery of project components, supported by project nurse practitioners (NPs). From this, it can be inferred that participants were familiar with the interviewers. In addition, participants were read a study information sheet during telephone contact prior to interview. Informed consent was received from each participant prior to interview. (Page 2) |
| 8. | Interviewer characteristics | What characteristics were reported about the interviewer/facilitator? e.g. *Bias, assumptions, reasons and interests in the research topic* | X/√ While the interviewer-authors did not report their biases or assumptions; however, the background section of the paper outlines the project's objectives, which can serve as a proxy for their interest in the topic. (Page 2) |
| **Domain 2: study design** | | |  |
| Theoretical framework | | |  |
| 9. | Methodological orientation and Theory | What methodological orientation was stated to underpin the study? *e.g. grounded theory, discourse analysis, ethnography, phenomenology, content analysis* | √ The authors describe that “*The method of analysis similar to the Framework Method as described by Gale et al [16] as an appropriate method to conduct content analysis within multidisciplinary teams”.* From this it can be deducted that the authors used content analysis. (Methods section, page 2) |
| Participant selection | | |  |
| 10. | Sampling | How were participants selected? *e.g. purposive, convenience, consecutive, snowball* | √ While no description of the sampling method was specifically provided by the authors, but it can be deducted that purposive sampling was applied to recruit participants. Participants were specifically identified by the larger, overarching OPTIMISTIC project. Project nurses in the OPTIMISTIC project specifically identified family members highly involved in the transfer decision of residents, indicating that the selection was based on specific criteria relevant to the project’s objectives. (Page 2) |
| 11. | Method of approach | How were participants approached? e*.g. face-to-face, telephone, mail, email* | √ Authors describe that interviews were conducted by telephone. (Page 2) |
| 12. | Sample size | How many participants were in the study? | √ Authors report that 20 participants took part in the project in the Abstract (Page 1) and Methods section (Page 2) |
| 13. | Non-participation | How many people refused to participate or dropped out? Reasons? | √ Authors report that “all identified family members agreed to participate in an interview” indicating zero refusals. No drop outs were reported. (Page 2) |
| Setting | | |  |
| 14. | Setting of data collection | Where was the data collected? e*.g. home, clinic, workplace* | √ Authors report that the data was collected via telephone, indicating the workplace as the setting. (Page 2) |
| 15. | Presence of non-participants | Was anyone else present besides the participants and researchers? | √ Authors report that the interviews were telephone based so it can be deducted that only the interviewer and participant were present. (Page 2) |
| 16. | Description of sample | What are the important characteristics of the sample? *e.g. demographic data, date* | **X/√** Authors report participant gender and their relationship to nursing home residents. No other demographic info provided. Interviews were conducted between Nov 2014 and March 2015. (Page 2) |
| Data collection | | |  |
| 17. | Interview guide | Were questions, prompts, guides provided by the authors? Was it pilot tested? | **√** Authors report that the interview consisted of 26 questions and prompts, and provided some example questions. (Page 2) |
| 18. | Repeat interviews | Were repeat interviews carried out? If yes, how many? | **√** Repeat interviews were not reported so it can be assumed that none were conducted. (Page 2) |
| 19. | Audio/visual recording | Did the research use audio or visual recording to collect the data? | **√** Although authors did not explicitly state that the interviews were recorded, it can be inferred that the telephone interviews were indeed recorded, as they report having obtained transcripts (Page 2). |
| 20. | Field notes | Were field notes made during and/or after the interview or focus group? | X Authors do not report if field notes were taken. |
| 21. | Duration | What was the duration of the interviews or focus group? | **√** Authors report that interviews were approx. 30 minutes long. (Page 2) |
| 22. | Data saturation | Was data saturation discussed? | X Authors do no discuss data saturation. |
| 23. | Transcripts returned | Were transcripts returned to participants for comment and/or correction? | **√** Authors report that interviews were not returned to participants (Page 2) |
| **Domain 3: analysis and findings** | | | |
| Data analysis | | | |
| 24. | Number of data coders | How many data coders coded the data? | **√** 4 authors coded and their initials reported (Page 3) |
| 25. | Description of the coding tree | Did authors provide a description of the coding tree? | **√** A table reporting themes and subthemes were provided. (Page 3) |
| 26. | Derivation of themes | Were themes identified in advance or derived from the data? | **√** Authors report that codes and themes were allowed to emerge from the data – inductive coding (Page 3) |
| 27. | Software | What software, if applicable, was used to manage the data? | X Not reported |
| 28. | Participant checking | Did participants provide feedback on the findings? | X Not reported |
| **Reporting** | | | |
| 29. | Quotations presented | Were participant quotations presented to illustrate the themes / findings? Was each quotation identified? e*.g. participant number* | **√** |
| 30. | Data and findings consistent | Was there consistency between the data presented and the findings? | **√** |
| 31. | Clarity of major themes | Were major themes clearly presented in the findings? | **√** |
| 32. | Clarity of minor themes | Is there a description of diverse cases or discussion of minor themes? | **√** The descriptive findings are presented based on Themes and sub themes. |

**Example 2:** Arendts et al (2015) “They never talked to me about…Perspectives on aged care resident transfer to emergency departments”

| **No** | **Item** | **Guide questions/description** | **Response/Page** |
| --- | --- | --- | --- |
| **Domain 1: Research team and reflexivity** | | |  |
| Personal Characteristics | | |  |
| 1. | Interviewer/facilitator | Which author/s conducted the interview or focus group? | √ The initials of authors who conducted the interviewers are reported in the method (Page 95) |
| 2. | Credentials | What were the researcher's credentials? *E.g. PhD, MD* | X Researchers’ credentials are not reported |
| 3. | Occupation | What was their occupation at the time of the study? | X Researchers’ occupations are not reported but their places of employment are provided. |
| 4. | Gender | Was the researcher male or female? | √ The gender of the authors can be assumed from their Christian names (ie 5 females and 1 male) (Page 95) |
| 5. | Experience and training | What experience or training did the researcher have? | X Researchers’ experience and training is not described. However, reflexivity was reported and facilitated by on-going dialogues with the authors throughout data collection and analysis. |
| Relationship with participants | | |  |
| 6. | Relationship established | Was a relationship established prior to study commencement? | X Authors did not report if any prior relationship was established prior to study commencement. (Page 95) |
| 7. | Participant knowledge of the interviewer | What did the participants know about the researcher? e*.g. personal goals, reasons for doing the research* | √ Authors indicate that participants were made fully aware of the reasons for the research prior to the interview (Page 96). |
| 8. | Interviewer characteristics | What characteristics were reported about the interviewer/facilitator? e.g. *Bias, assumptions, reasons and interests in the research topic* | X/√ While the interviewer-authors did not report their biases or assumptions; however, the background section of the paper outlines the project's objectives, which can serve as a proxy for their interest in the topic. (Page 95) |
| **Domain 2: study design** | | |  |
| Theoretical framework | | |  |
| 9. | Methodological orientation and Theory | What methodological orientation was stated to underpin the study? *e.g. grounded theory, discourse analysis, ethnography, phenomenology, content analysis* | √ The authors did not describe the methodological orientation, but it can be deducted from the project focus/aim that phenomenology underpinned the project. (Page 95) |
| Participant selection | | |  |
| 10. | Sampling | How were participants selected? *e.g. purposive, convenience, consecutive, snowball* | √ Authors report that sampling was purposive (Page 96) |
| 11. | Method of approach | How were participants approached? e*.g. face-to-face, telephone, mail, email* | √ Authors describe that residents & relatives were approached in-person by research staff in the ED (Page 96) |
| 12. | Sample size | How many participants were in the study? | √ Authors report that 11 residents and 14 relatives took part (Page 95 & 96) |
| 13. | Non-participation | How many people refused to participate or dropped out? Reasons? | X Not reported |
| Setting | | |  |
| 14. | Setting of data collection | Where was the data collected? e*.g. home, clinic, workplace* | √ Authors report that the data was collected at a “time and place of respondents choosing which indicates the nursing home or other place of residence. (Page 95) |
| 15. | Presence of non-participants | Was anyone else present besides the participants and researchers? | √ Not reported but it can be deducted that only the interviewer and participant were present. (Page 96) |
| 16. | Description of sample | What are the important characteristics of the sample? *e.g. demographic data, date* | **√** Authors report participant gender, age. No info provided on dates (Page 96) |
| Data collection | | |  |
| 17. | Interview guide | Were questions, prompts, guides provided by the authors? Was it pilot tested? | **√** Authors provide copy of the interview schedule (Page 102) |
| 18. | Repeat interviews | Were repeat interviews carried out? If yes, how many? | **√** Repeat interviews were not reported so it can be assumed that none were conducted. (Page 96) |
| 19. | Audio/visual recording | Did the research use audio or visual recording to collect the data? | **√** Although authors stated that the interviews were recorded (Page 96). |
| 20. | Field notes | Were field notes made during and/or after the interview or focus group? | **√**  Authors report that field notes were taken. (Page 96) |
| 21. | Duration | What was the duration of the interviews or focus group? | **√** Authors did not report that interview time |
| 22. | Data saturation | Was data saturation discussed? | **√** Authors do discuss data saturation. (Page 96) |
| 23. | Transcripts returned | Were transcripts returned to participants for comment and/or correction? | **X** Authors did not report that interviews were not returned to participants |
| **Domain 3: analysis and findings** | | | |
| Data analysis | | | |
| 24. | Number of data coders | How many data coders coded the data? | **√** 2 authors coded and their initials reported (Page 96) |
| 25. | Description of the coding tree | Did authors provide a description of the coding tree? | **√** Authors reported development of coding tree with nodes based on interview content and coded transcripts. (Page 96) |
| 26. | Derivation of themes | Were themes identified in advance or derived from the data? | **√** Authors report that initial coding framework was a conceptual model based on hypothesis derived from a prior literature review - deductive coding (Page 96) |
| 27. | Software | What software, if applicable, was used to manage the data? | **√** NVivo (Page 96) |
| 28. | Participant checking | Did participants provide feedback on the findings? | X Not reported |
| **Reporting** | | | |
| 29. | Quotations presented | Were participant quotations presented to illustrate the themes / findings? Was each quotation identified? e*.g. participant number* | **√** |
| 30. | Data and findings consistent | Was there consistency between the data presented and the findings? | **√** |
| 31. | Clarity of major themes | Were major themes clearly presented in the findings? | **√** |
| 32. | Clarity of minor themes | Is there a description of diverse cases or discussion of minor themes? | **√** The descriptive findings are presented based on themes and subthemes. |
